# Public Awareness of and Personal Willingness to Use California's Extreme Risk Protection Order Law to Prevent Firearm-Related Harm

**DOI:** 10.1001/jamahealthforum.2021.0975

**Published:** 2021-06-04

**Authors:** Nicole Kravitz-Wirtz, Amanda J. Aubel, Rocco Pallin, Garen J. Wintemute

**Affiliations:** 1 University of California Firearm Violence Research Center and Violence Prevention Research Program, Department of Emergency Medicine, University of California Davis School of Medicine, Sacramento

## Abstract

**Importance:**

Extreme risk protection order (ERPO) laws temporarily suspend firearm and ammunition access by individuals whom a judge has deemed to be at substantial risk of harming themselves or others. Despite widespread recent adoption of these laws, use of ERPOs has been limited. Barriers to ERPO uptake remain unclear.

**Objective:**

To assess public awareness and perceived appropriateness of and willingness to use ERPOs in various risk scenarios, and to identify reasons for being unwilling, overall and by firearm ownership status, to inform efforts to improve ERPO implementation.

**Design, Setting, and Participants:**

This was a cross-sectional study using data from the 2020 California Safety and Wellbeing Survey, a statewide internet survey on firearm ownership and exposure to violence and its consequences, conducted from July 14 to July 27, 2020. Adult respondents were recruited from the Ipsos KnowledgePanel using probability-based sampling methods. Responses were weighted to be representative of the adult population of California.

**Main Outcomes and Measures:**

Awareness and perceived appropriateness of gun violence restraining orders (GVROs; California’s official term for ERPOs), willingness to use a GVRO for a family member at risk of harm, and reasons for being not at all willing to use a GVRO in 1 or more risk scenarios, overall and by firearm ownership status.

**Results:**

Of the 5018 panel members invited, 2870 (57%) completed the survey. Of these respondents (mean [SD] age: 47.9 [16.9] years; 52.3% women; 41.9% White, 34.7% Latinx, 14.4% Asian, and 5.8% Black individuals), 65.6% (95% CI, 63.0%-68.1) had never heard of a GVRO or a red flag law. Firearm owners were significantly more likely (20.5%; 95% CI, 15.9%-26.0%) than nonowners who live with owners (6.1%; 95% CI, 3.7%-10.0%; *P* < .001) and nonowners (9.6%; 95% CI, 7.8%-11.6%; *P* < .001) to have heard of both a GVRO and a red flag law. After reading a brief description of California’s GVRO law, 72.9% (95% CI, 70.2%-75.4%) to 78.4% (95% CI, 75.9%-80.8%) of respondents, depending on the risk scenario, indicated that GVROs were in general at least sometimes appropriate, while 73.2% (95% CI, 70.5%-75.6%) to 83.6% (95% CI, 81.2%-85.8%) said they would be somewhat or very willing to use a GVRO for a family member at risk of harm. Firearm owners reported the highest levels of GVRO appropriateness in 4 of 5 risk scenarios (depending on the scenario, 80.0% [95% CI, 73.6%-85.1%] to 85.6% [95% CI, 79.9%-89.8%]). Nonowners who live with owners reported the highest levels of personal willingness to use a GVRO (depending on the scenario, 83.7% [95% CI, 74.7%-90.0%] to 94.7% [95% CI, 86.2%-98.1%]). The most frequently cited reasons for being unwilling to use a GVRO were not knowing enough about GVROs (44.9%; 95% CI, 39.7%-50.3%), believing the risk scenarios are personal or family matters (26.6%; 95% CI, 22.2%-31.6%), and distrust that the system will be fair (23.1%; 95% CI, 19.1%-27.6%).

**Conclusions and Relevance:**

In this cross-sectional survey study, public awareness of GVROs was low, but perceived appropriateness of and willingness to use these tools at least some of the time was high. Foci for efforts to address barriers to GVRO use in California were identified; similar challenges likely exist in other jurisdictions.

## Key Points

**Questions:**

What does the public know about extreme risk protection orders (ERPOs) and are people willing to use these tools to prevent firearm-related harm, both in general and when a family member is at risk? If they are not willing, why not?

**Findings:**

In this state-representative cross-sectional survey study of 2870 adults in California, support for the appropriateness of and willingness to use an ERPO at least some of the time was high (>70%) across selected risk scenarios, particularly among firearm owners and nonowners who live with owners. Approximately two-thirds of respondents cited a lack of awareness of ERPOs as a reason why they were unwilling to use an EPRO, followed by a belief that the risk scenarios are private matters, and a distrust that the system will be fair.

**Meaning:**

These findings on public awareness and perceived appropriateness of ERPOs, willingness to use these tools to prevent firearm-related harm, and areas of focus to address barriers to EPRO uptake may be informative for EPRO implementation in California and other jurisdictions.

## Teaser

This cross-sectional study assesses public awareness and perceived appropriateness of using extreme risk protection orders in various risk scenarios and identifies reasons behind reluctance, overall and by firearm ownership status.

## Introduction

Firearms are involved in more than half of suicides and approximately three-quarters of homicides in the US, accounting for nearly 39 000 violent deaths in 2018.^1^ While firearm violence is a vast public health problem, it is not inevitable. Policies and programs designed to prevent firearm-related harm are operating or are under consideration in jurisdictions across the country.^2^ Extreme risk protection order (ERPO) laws, sometimes referred to as “red flag” laws, have gained traction since the February 2018 mass shooting at Marjory Stoneman Douglas High School in Parkland, Florida, which many stakeholders believed could have been prevented had an ERPO been available.^3,4^ Extreme risk protection orders are targeted, flexible, and proactive tools for temporarily suspending an individual’s access to firearms and ammunition when a judge has deemed the person to be at increased risk of firearm-related harm to themself or others and they are not otherwise prohibited from firearm ownership. Immediate family, household members, and law enforcement officers are typically permitted to petition for an EPRO.^5,6^

Legislation on ERPOs has expanded rapidly. Of the 19 states and the District of Columbia with ERPO-type laws in effect as of February 2021, all but 2 were implemented since 2016. Yet early evidence suggests that use of ERPOs remains relatively uncommon.^7,8^ There are various possible reasons for these lags in uptake. Central among these are questions about the extent to which the public is (1) aware of ERPOs, and (2) willing to use them in efforts to prevent firearm-related harm. Additionally, while ERPO laws have received greater bipartisan support^9^ and less pushback from gun rights organizations than many firearm violence prevention policies—eg, the National Rifle Association lobbied Congress to provide funding for states to adopt ERPO laws in the aftermath of the Parkland shooting—the perception that public support is deeply divided by gun ownership status has still been characterized as a speedbump on the road to ERPO implementation in many jurisdictions.^10^

The present study examines public awareness of and personal willingness to use an ERPO in specific risk scenarios, both in general and when a hypothetical family member is at risk, along with reasons for unwillingness to use such a tool, overall and by gun ownership status. We focused on California, which implemented the first ERPO law in January 2016 (2 states had related "risk warrant" laws in effect previously). In California, ERPOs are termed gun violence restraining orders (GVROs). As in other states, use of GVROs has increased over time in California, from 70 orders in 2016 to 700 in 2019; however, overall uptake of the law has been slow, with the increase driven primarily by law enforcement petitioners in 1 county.^7^ With renewed interest in short-term crisis interventions to prevent firearm injury and death amid the COVID-19 pandemic—at a time when firearm purchasing has increased and conditions that elevate the risk of harm have worsened^11,12,13,14,15^—this study is intended to identify opportunities to improve messaging and increase uptake of GVROs. As an early adopter and model for subsequent legislation in other states, we view the California experience as broadly informative for ERPO implementation in other jurisdictions.

## Methods

This was a cross-sectional survey study using data from the 2020 California Safety and Wellbeing Survey, a statewide survey on firearm ownership and exposure to violence and its consequences. The survey was designed by the University of California Firearm Violence Research Center and the Violence Prevention Research Program, both at the University of California, Davis, and administered online from July 14 to July 27, 2020, by Ipsos SA (Paris). The survey was approved by the University of California Davis Institutional Review Board. Respondents read informed consent language; study initiation constituted consent. This study followed the American Association for Public Opinion Research (AAPOR) reporting guideline.

Survey respondents were recruited from the Ipsos KnowledgePanel, an online research panel widely used in health-related research.^16,17,18,19^ Panel recruitment using probability-based sampling procedures has been described previously.^11^ All panel members who were 18 years or older and residents of California, except those currently serving in the US Armed Forces, were eligible to participate. Invitations were sent by email; automatic reminders were emailed to nonresponders 3 days later. Of 5018 members invited to participate, 2870 completed the survey (completion rate, 57%). The median completion time was 26 minutes.

A final weight variable provided by Ipsos adjusted for the initial probability of panel selection and for survey-specific nonresponse and over-coverage or under-coverage using poststratification raking ratio adjustments based on cross-classifications of age, gender, race/ethnicity, education, household income, language proficiency, and California region. For race/ethnicity, respondents were asked if they consider themselves to be Latinx (Hispanic, Latino, or Spanish; yes/no) and to select their race(s): American Indian/Alaska Native, Asian, Black/African American, Native Hawaiian/other Pacific Islander, and/or White. Respondents tended to be older, male, non–Latinx, and have more years of education and higher income than nonrespondents.^11^ The sample was weighted to be statistically representative of the adult population of California, as reflected in the 2018 American Community Survey.^20^

### Measures

Respondents were asked whether they had ever heard of something called a GVRO, and subsequently, a red flag law. Next, respondents read a description of California’s GVRO law and were asked, “In general, do you think it would be appropriate for a judge to issue a GVRO” in each of 5 risk scenarios: when a person (1) is experiencing an emotional crisis, (2) has severe dementia or something like it, (3) has threatened to physically hurt themselves, (4) has threatened to physically hurt someone else, and (5) has threatened to physically hurt a group of people. Respondents could choose never, sometimes, usually, always, or don’t know. Those who answered “don’t know” were asked whether this was because they needed more information about GVROs.

Respondents were then asked, “Would *you personally* be willing to ask a judge for a GVRO if a member of your family” was in each of the 5 risk scenarios. Respondents could choose not at all, somewhat, or very willing. A follow-up question asked whether respondents "would prefer to have the police ask a judge for a GVRO for you." Those who reported being “not at all” willing to ask a judge for a GVRO in 1 or more scenarios were asked about the reasons for their unwillingness.

Respondents were grouped by firearm ownership status: firearm owners, nonowners who live with owners, and nonowners in households without firearms (hereafter, nonowners). Additional items of interest included whether respondents reported knowing anyone who had experienced or was at risk of harm to self or others, as well as sociodemographic information, which was collected separately as part of ongoing panel membership and was merged with study responses. Sociodemographic characteristics of respondents, overall and by firearm ownership status, are presented in Table 1.

**Table 1.** Sociodemographic Characteristics of Respondents, by Firearm Ownership Status, 2020 California Safety and Wellbeing Survey^a^

Characteristic	Nonowners (n = 1989)	Firearm owners (n = 529)	Nonowners who live with owners (n = 219)	Total (n = 2870)
	No. (%) [95% CI]	No. (%) [95% CI]	No. (%) [95% CI]	No. (%) [95% CI]
Age, y				
18-29	156 (17.3) [14.6-20.5]	12 (8.1) [4.4-14.6]	23 (24.6) [16.0-35.9]	200 (16.3) [14.0-18.9]
30-44	387 (31.2) [28.0-34.6]	54 (25.1) [18.6-33.0]	40 (20.4) [13.9-29.0]	511 (29.7) [27.0-32.5]
45-59	517 (26.4) [23.7-29.4]	130 (29.1) [23.5-35.5]	50 (23.7) [16.6-32.7]	738 (26.7) [24.4-29.2]
60+	929 (25) [22.8-27.5]	333 (37.6) [32.1-43.5]	106 (31.3) [23.6-40.0]	1421 (27.3) [25.3-29.4]
Sex				
Female	1010 (54.8) [51.5-58.1]	126 (26.5) [21.3-32.3]	166 (74.9) [64.8-82.9]	1364 (52.3) [49.5-55.0]
Male	979 (45.2) [41.9-48.5]	403 (73.5) [67.7-78.7]	53 (25.1) [17.1-35.2]	1506 (47.7) [45.0-50.5]
Race/ethnicity^b^				
White	1035 (36.7) [33.7-39.8]	373 (60.2) [53.1-67.0]	141 (55.8) [45.7-65.3]	1615 (41.9) [39.3-44.6]
Latinx	653 (38.1) [34.9-41.5]	97 (22.2) [16.6-29.0]	54 (22.9) [15.9-31.9]	849 (34.7) [32.0-37.4]
Asian	169 (16.7) [14.1-19.7]	22 (10.4) [6.2-16.9]	7 (6.1) [2.6-13.9]	208 (14.4) [12.3-16.8
Black	86 (5.7) [4.3-7.5]	26 (6.0) [3.4-10.2]	7 (4.8) [1.7-12.8]	127 (5.8) [4.6-7.3]
Multirace/other	46 (2.8) [1.8-4.2]	11 (1.2) [0.6-2.6]	10 (10.4) [4.9-20.9]	71 (3.2) [2.3-4.5]
Education				
<High school	137 (18.6) [15.7-21.8]	6 (3.5) [1.4-8.1]	8 (10.3) [4.8-20.5]	158 (15.4) [13.1-18.0]
High school degree	253 (19.4) [16.7-22.3]	64 (24.7) [18.5-32.1]	26 (20.6) [12.9-31.3]	364 (20.4) [18.1-23.0]
Some college	581 (28.1) [25.3-31.0]	213 (44.6) [38.1-51.3]	80 (41.2) [32.0-51.0]	938 (32.7) [30.2-35.2]
College degree	558 (18.7) [16.5-21.1]	137 (16.5) [12.3-21.7]	57 (14.6) [10.2-20.6]	775 (17.5) [15.7-19.4]
Advanced degree	460 (15.3) [13.2-17.7]	109 (10.8) [8.3-14.1]	48 (13.4) [8.9-19.6]	635 (14.0) [12.4-15.8]
Household income, $				
<25 000	334 (16.8) [14.4-19.4]	52 (8.3) [5.3-12.8]	19 (8.4) [4.6-14.9]	431 (15.1) [13.2-17.2]
25 000-59 999	539 (26.0) [23.3-29.0]	128 (19) [14.8-24.0]	45 (17.8) [11.5-26.4]	744 (23.9) [21.7-26.3]
60 000-99 999	523 (23.1) [20.4-26.0]	132 (19.1) [14.8-24.2]	60 (20.9) [14.8-28.7]	757 (22.6) [20.4-24.9]
≥100 000	593 (34.1) [31.0-37.4]	217 (53.6) [46.9-60.1]	95 (52.9) [43.2-62.3]	938 (38.5) [35.8-41.3]
Marital status				
Married or living with partner	1123 (59.2) [55.9-62.4]	344 (66.7) [60.1-72.7]	156 (64.3) [54.1-73.4]	1704 (61.0) [58.2-63.7]
Widowed	150 (4.4) [3.5-5.5]	39 (5.7) [3.6-8.9]	9 (3.8) [1.6-8.9]	204 (4.4) [3.6-5.3]
Divorced or separated	302 (10.8) [9.1-12.7]	89 (13.6) [9.7-18.8]	22 (7.3) [4.4-12.1]	432 (11.1) [9.6-12.7]
Never married	414 (25.6) [22.6-28.8]	57 (13.9) [9.6-19.8]	32 (24.6) [16.3-35.3]	530 (23.5) [21.1-26.1]
Political ideology				
Liberal	738 (34.2) [31.1-37.4]	109 (20.5) [15.6-26.4]	68 (31.3) [23.1-40.9]	940 (31.1) [28.6]-33.7
Moderate	551 (30.5) [27.5-33.7]	143 (28.5) [22.7-35.2]	69 (36.5) [27.5-46.6]	795 (30.8) [28.2-33.5]
Conservative	511 (23.5) [20.9-26.4]	245 (43.3) [36.8-50.1]	66 (24.4) [17.5-33.0]	882 (27.3) [25.0-29.8]
State region				
Superior California	142 (6.7) [5.2-8.5]	79 (15.2) [10.6-21.2]	20 (5.8) [3.2-10.3]	253 (8.1) [6.7-9.7]
North Coast	47 (2.2) [1.3-3.5]	32 (4.8) [3.0-7.5]	9 (2.3) [1.1-4.9]	90 (2.5) [1.8-3.5]
San Francisco Bay Area	396 (20.9) [18.1-24.1]	75 (11.8) [8.2-16.5]	25 (14.8) [8.7-24.1]	513 (18.5) [16.3-21.0]
Northern San Joaquin Valley	69 (3.6) [2.6-5.1]	35 (7.6) [4.8-11.8]	15 (4.4) [2.2-8.4]	129 (4.5) [3.5-5.7]
Central Coast	143 (6.0) [4.8-7.4]	38 (4.9) [3.2-7.4]	19 (13.3) [7.3-22.9]	204 (6.2) [5.1-7.5]
Southern San Joaquin Valley	116 (5.4) [4.2-7.0]	34 (7.1) [4.1-12.3]	20 (7.8) [4.3-13.6]	182 (6.0) [4.8-7.4]
Inland Empire	225 (10.4) [8.6-12.5]	74 (13.6) [9.7-18.6]	31 (8.5) [5.2-13.5]	358 (11.0) [9.5-12.8]
Los Angeles County	500 (27.1) [24.4-30.1]	74 (18.1) [13.4-24.1]	50 (28) [20.1-37.5]	647 (25.9) [23.6-28.4]
Orange County	137 (8.7) [6.9-10.8]	38 (7.5) [4.5-12.1]	10 (7.2) [2.9-16.7]	195 (8.3) [6.8-10.0]
San Diego–Imperial	214 (9.0) [7.4-11.0]	50 (9.5) [6.0-14.7]	20 (8.0) [4.0-15.6]	299 (9.1) [7.7-10.8]

^a^ Totals may not sum to 100% because refusals and “don't know” responses are not shown.

^b^ Respondents were asked, “Are you Spanish, Hispanic, or Latino? Indicate what you consider your race to be. We appreciate your effort to describe your background using these US Census Bureau categories (select all that apply): American Indian/Alaska Native, Asian, Black/African American, Native Hawaiian/other Pacific Islander, and White.” Among respondents who did not endorse Spanish, Hispanic, or Latino ethnicity, American Indian or Alaska Native (n = 10), Native Hawaiian or other Pacific Islander (n = 2), and those who selected more than 1 race (n = 59) were combined by the authors into a multirace/other category.

The original description of California’s GVRO law was followed by 1 of 3 randomized statements about the research evidence on EPRO effectiveness or by a no-statement control. No substantive differences were observed across the 4 conditions; therefore, results reflect the full sample, regardless of randomization. To align with GVRO statute language, questions about GVRO appropriateness and willingness included the qualifier: “Assume [the person/your family member] has or could get a gun and other options have failed or are not appropriate.” Detailed question phrasing and response options are provided in the eMethods section of the Supplement.

### Statistical Analysis

We calculated weighted percentages and 95% CIs for each measure or cross-tabulation of measures in Stata, release 15.1 (StataCorp LLC) using the survey and weighting commands. Comparisons by firearm ownership status were conducted using the 2-sample *t* test within the framework of the weighted population. For ease of exposition and consistent with past research,^16^ we collapsed “sometimes,” “usually,” and “always” appropriate and “somewhat” and “very” willing into single response categories for primary analyses; analogous results with all response options are available in eTables 1A and 1B of the Supplement. Analyses were conducted from October 2020 to February 2021. All statistical tests were 2-tailed, and statistical significance was set at *P* < .05.

## Results

### Awareness

Of 2870 respondents, (mean [SD] age was 47.9 [16.9] years and 52.3% were women; 49.1% were White, 34.7% Latinx, 14.4% Asian, and 5.8% Black individuals), 65.6% (95% CI, 63.0%-68.1%) reported that they had never heard of a GVRO or a red flag law; 11.0% (95% CI, 9.5%-12.8%) had heard of both; 13.9% (95% CI, 12.1%-15.8%) had heard of a GVRO only; and 8.8% (95% CI, 7.5%-10.4%) had heard of a red flag law only (Table 2). Firearm owners were significantly more likely (20.5%) than nonowners who live with owners (6.1%; *P* < .001) and nonowners (9.6%; *P* < .001) to have heard of both a GVRO and a red flag law, and significantly less likely to never have heard of either (51.6% vs 70.5% [*P* = .001] and 68.2% [*P* < .001], respectively) (Table 2). Firearm owners were also significantly more likely than nonowners (14.9% vs 7.3%; *P* = .004; Table 2) to have heard of a red flag law only.

**Table 2.** Public Awareness of Gun Violence Restraining Orders (GVROs) and “Red Flag” Laws Among California Adults, by Firearm Ownership Status, 2020 California Safety and Wellbeing Survey^a^

Awareness status^**b**^	Total (n = 2870)		Firearm ownership status								
			Nonowners (n = 1989)			Firearm owners (n = 529)			Nonowners who live with owners (n = 219)		
	No.	% (95% CI)	No.	% (95% CI)	*P* value	No.	% (95% CI)	*P* value	No.	% (95% CI)	*P* value
Yes, GVRO only	464	13.9 (12.1-15.8)	322	14.4 (12.3-16.8)	NS	88	13.0 (9.5-17.5)	NS	39	15.0 (9.0-23.8)	NS
Yes, red flag law only	275	8.8 (7.5-10.4)	162	7.3 (5.9-9.0)	.004^c^	75	14.9 (10.6-20.4)	.004^d^	20	8.4 (4.4-15.5)	NS
Yes, both GVRO and red flag law	427	11.0 (9.5-12.8)	258	9.6 (7.8-11.6)	<.001^c^	128	20.5 (15.9-26.0)	<.001^d^	24	6.1 (3.7-10.0)	<.001^c^
								<.001^e^			
Neither GVRO nor red flag	1688	65.6 (63.0-68.1)	1238	68.2 (65.1-71.2)	<.001^c^	237	51.6 (44.8-58.3)	<.001^d^	136	70.5 (61.1-78.5)	.001^c^
								.001^e^			

Abbreviation: NS, not significant.

^a^ Percentages may not sum to 100% because refusals are not shown: 0.7% (95% CI, 0.4-1.3) of respondents did not answer 1 or both questions (n = 16).

^b^ Respondents were asked, “Have you ever heard of something called a gun violence restraining order?” and “Have you ever heard of something called a 'red flag' law?”

^c^ Significant difference from firearm owners.

^d^ Significant difference from nonowners.

^e^ Significant difference from nonowners who live with owners.

### Appropriateness

More than three-quarters of respondents indicated that, in general, it was at least sometimes appropriate for a judge to issue a GVRO when the at-risk person has threatened to physically hurt a group of people (78.4%; 95% CI, 75.9%-80.8%), someone else (78.1%; 95% CI, 75.5%-80.8%), or themselves (77.8%; 95% CI, 75.3%-80.2%) (Table 3). A slightly smaller share of respondents indicated that, in general, a GVRO is at least sometimes appropriate when the person is experiencing an emotional crisis (73.0%; 95% CI, 70.3%-75.4%) or has severe dementia or something like it (72.9%; 95% CI, 70.2%-75.4%). Of the 19.1% (95% CI, 16.9%-21.6%) of respondents who reported not knowing whether issuing a GVRO was appropriate in 1 or more scenarios, 48.5% (95% CI, 41.8%-55.3%) said that this was because they needed more information about GVROs (eTable 2 in the Supplement).

**Table 3.** Perceived Appropriateness of a Judge Issuing a Gun Violence Restraining Orders (GVRO), in General, by Risk Scenario and Firearm Ownership Status, 2020 California Safety and Wellbeing Survey (n = 2870)^a^

Risk scenario and firearm ownership status	Appropriate to issue GVRO^**b**^				*P* value
	Never		Sometimes/usually/always		
	No.	% (95% CI)	No.	% (95% CI)	
Person experiencing an emotional crisis					
Total	296	11.5 (9.9-13.5)	2203	73.0 (70.3-75.4)	NS
Nonowners	182	11.3 (9.3-1.7)	1546	72.2 (69.0-75.2)	.01^c^
Firearm owners	76	15.0 (10.9-20.3)	410	75.2 (68.8-80.6)	NS
Nonowners who live with owners	15	6.7 (2.9-14.7)	178	82.2 (73.6-88.4)	.01^d^
Person with severe dementia or something like it					
Total	286	12.2 (10.4-14.2)	2253	72.9 (70.2-75.4)	NS
Nonowners	198	12.3 (10.2-14.8)	1559	71.8 (68.5-74.8)	.01^e^
Firearm owners	50	10.7 (7.2-15.6)	440	80.0 (73.6-85.1)	.014^d^
Nonowners who live with owners	19	13.4 (7.1-23.6)	180	76.3 (66.2-84.1)	NS
Person who threatened to physically hurt themself					
Total	235	11.3 (9.5-13.3)	2396	77.8 (75.3-80.2)	NS
Nonowners	162	11.2 (9.2-13.7)	1661	77.0 (73.8-79.8)	.02^e^
Firearm owners	40	11.8 (7.8-17.4)	466	84.1 (78.4-88.6)	.02^d^
Nonowners who live with owners	16	9.4 (4.5-18.8)	187	80.7 (71.0-87.7)	NS
Person who threatened to physically hurt someone else					
Total	235	11.3 (9.5-13.3)	2413	78.1 (75.5-80.5)	NS
Nonowners	169	11.9 (9.8-14.4)	1662	76.5 (73.3-79.5)	.002^e^
Firearm owners	36	9.5 (6.2-14.3)	474	85.6 (79.9-89.8)	.002^d^
Nonowners who live with owners	15	8.9 (4.1-18.4)	190	83.4 (73.9-89.8)	NS
Person who threatened to physically hurt a group of people					
Total	238	11.7 (9.9-13.8)	2415	78.4 (75.9-80.8)	NS
Nonowners	172	12.4 (10.2-15.0)	1669	77.5 (74.4-80.3)	.008^e^
Firearm owners	36	9.8 (6.2-15.2)	471	85.3 (79.6-89.6)	.008^d^
Nonowners who live with owners	15	9.3 (4.4-18.7)	190	81.5 (71.8-88.4)	NS

Abbreviation: NS, not significant.

^a^ Percentages may not sum to 100% because refusals and “don't know” responses are not shown.

^b^ Respondents were asked, “In general, do you think it would be appropriate for a judge to issue a GVRO in the following scenarios? Assume the person has or could get a gun and other options have failed or are not appropriate.”

^c^ Significant difference from nonowners who live with owners.

^d^ Significant difference from nonowners.

^e^ Significant difference from firearm owners.

In 4 of 5 risk scenarios, firearm owners reported the highest levels of at least some support—80% or more—for the appropriateness of GVROs in general (Table 3). Firearm owners were significantly more likely than nonowners to indicate that, in general, it is at least sometimes appropriate for a judge to issue a GVRO when the at-risk person has severe dementia or something like it (80.0% vs 71.8%; *P* = .01); or has threatened to physically hurt themselves (84.1% vs 77.0%; *P* = .02), someone else (85.6% vs 76.5%; *P* = .002), or a group of people (85.3% vs 77.5%; *P* = .008). Nonowners who live with owners were significantly more likely than nonowners (82.2% vs 72.2%; *P* = .01) to report that, in general, GVROs are at least sometimes appropriate when the person is experiencing an emotional crisis.

### Willingness

Most respondents indicated that they would be somewhat or very willing to ask a judge for a GVRO if a family member was experiencing an emotional crisis (73.2%; 95% CI, 70.5%-75.6%); had severe dementia or something like it (76.5%; 95% CI, 73.9%-78.9%); or had threatened to physically hurt themself (82.8%; 95% CI, 80.4%-85.0%), someone else (83.8%; 95% CI, 81.4%-85.9%), or a group of people (83.6%; 95% CI, 81.2%-85.8%) (Table 4). Knowing someone who had experienced or was at risk of harm to self was associated with significantly higher levels of at least some willingness to use a GVRO in the harm-to-self scenario; however, knowing someone who had experienced or was at risk of interpersonal harm was not associated with differences in willingness in the harm-to-others scenarios (eTables 3A-3F in the Supplement).

**Table 4.** Willingness to Ask a Judge for a Gun Violence Restraining Order (GVRO) for a Family Member, by Risk Scenario and Firearm Ownership Status, 2020 California Safety and Wellbeing Survey (n = 2870)^a^

Risk scenario and firearm ownership status	Willingness to ask for a GVRO^**b**^				*P* value
	Not at all		Somewhat/very		
	No.	% (95% CI)	No.	% (95% CI)	
Family member experiencing an emotional crisis					
Total	651	24.4 (22.0-27.0)	2168	73.2 (70.5-75.6)	NS
Nonowners	406	24.2 (21.3-27.3)	1548	73.1 (69.9-76.1)	.01^c^
Firearm owners	161	29.8 (23.9-36.4)	365	69.8 (63.2-75.7)	.006^c^
Nonowners who live with owners	38	16.3 (10.0-25.3)	181	83.7 (74.7-90.0)	.01^d^
					.006^e^
Family member with severe dementia or something like it					
Total	515	20.6 (18.4-23.1)	2290	76.5 (73.9-78.9)	NS
Nonowners	348	20.9 (18.2-23.9)	1597	76.1 (73.0-79.0)	NS
Firearm owners	103	21.5 (16.27-27.9)	421	78.0 (71.6-83.2)	NS
Nonowners who live with owners	25	15.3 (8.9-25.0)	193	84.5 (74.9-90.9)	NS
Family member who threatened to physically hurt themselves					
Total	318	14.3 (12.3-16.5)	2493	82.8 (80.4-85.0)	NS
Nonowners	207	14.3 (11.9-17.0)	1740	82.4 (79.5-85.0)	<.001^c^
Firearm owners	68	16.6 (12.0-22.6)	458	83.0 (77.0-87.7)	.003^c^
Nonowners who live with owners	9	5.4 (1.9-14.5)	210	94.6 (85.5-98.1)	<.001^d^
					.003^e^
Family member who threatened to physically hurt you or someone else					
Total	280	13.4 (11.4-15.6)	2529	83.8 (81.4-85.9)	NS
Nonowners	188	14.1 (11.7-16.9)	1758	82.6 (79.7-85.2)	<.001^c^
Firearm owners	57	14.2 (9.8-20.1)	470	85.6 (79.7-90.0)	.02^c^
Nonowners who live with owners	9	5.1 (1.8-13.8)	209	94.7 (86.2-98.1)	<.001^d^
					.02^e^
Family member who threatened to physically hurt a group of people					
Total	284	13.7 (11.7-16.0)	2533	83.6 (81.2-85.8)	NS
Nonowners	192	14.4 (12.0-17.3)	1761	82.6 (79.6-85.2)	.002^c^
Firearm owners	55	14.0 (9.8-20.0)	470	85.4 (79.4-89.9)	NS
Nonowners who live with owners	11	7.0 (2.9-15.8)	208	93.0 (84.2-97.1)	.002^d^

Abbreviation: NS, not significant.

^a^ Percentages may not sum to 100% because refusals are not shown.

^b^ Respondents were asked, “Would you personally be willing to ask a judge for a GVRO if a member of your family was in one of the following scenarios? Assume the person has or could get a gun and other options have failed or are not appropriate.”

^c^ Significant difference from nonowners who live with owners.

^d^ Significant difference from nonowners.

^e^ Significant difference from firearm owners.

Nonowners who live with owners reported the highest levels of at least some willingness to ask a judge for a GVRO across all risk scenarios, ranging from 83.7% (95% CI, 74.7%-90.0%) who were somewhat or very willing if a family member were experiencing an emotional crisis to 94.7% (95% CI, 86.2%-98.1%) if a family member had threatened to physically hurt someone else (Table 4). Differences in willingness between nonowners who live with owners and both firearm owners and nonowners were statistically significant (*P* < .05; Table 4) in all scenarios except dementia and mass harm, where only the contrast with nonowners was significant (*P* < .01; Table 4).

### Lack of Willingness

In all, 29.9% (95% CI, 27.3%-32.5%) of respondents reported that they were not at all willing to ask a judge for a GVRO if a family member were in 1 or more risk scenarios. The most frequently cited reason for being unwilling was not knowing enough about GVROs (44.9%; 95% CI, 39.7%-50.3%; Table 5); it was followed by the belief that these risk scenarios are personal or family matters (26.6%; 95% CI, 22.2%-31.6%), distrust that the system will be fair (23.1%; 95% CI, 19.1%-27.6%), worry about due process rights (18.6%; 95% CI, 15.3%-22.3%), not wanting to involve the court (16.4%; 95% CI, 12.7%-20.8%), the notion that it is never appropriate for the government to take a person’s guns (13.7%; 95% CI, 10.4%-17.9%), and worry about retaliation (11.3%; 95% CI, 8.4%-15.0%).

**Table 5.** Reasons Not At All Willing to Ask a Judge for a Gun Violence Restraining Order (GVRO) for a Family Member, by Firearm Ownership Status, 2020 California Safety and Wellbeing Survey^a^

Reason^b^	Total (n = 799)		Nonowners (n = 518)			Firearm owners (n = 181)			Nonowners who live with owners (n = 43)		
	No.	% (95% CI)	No.	% (95% CI)	*P* value	No.	% (95% CI)	*P* value	No.	% (95% CI)	*P* value
Do not know enough about GVROs	322	44.9 (39.7-50.3)	240	47.8 (41.4-54.3)	.04^c^	43	33.9 (23.4-46.3)	.04^d^	16	46.4 (25.4-68.7)	NS
These are personal/family matters	233	26.6 (22.2-31.6)	148	25.5 (20.4-31.5)	NS	57	29.2 (19.7-41.0)	NS	17	47.1 (26.1-69.2)	NS
Do not trust the system will be fair	218	23.1 (19.1-27.6)	119	20.1 (15.6-25.4)	.02^c^	71	34.0 (24.6-45.0)	.02^d^	9	23.0 (8.0-50.9)	NS
Worried about due process rights	201	18.6 (15.3-22.3)	109	14.5 (11.1-18.6)	.006^c^	65	27.7 (19.9-37.2)	.006^d^	11	35.3 (16.9-59.3)	NS
Do not want to involve the court	119	16.4 (12.7-20.8)	78	17.6 (12.9-23.4)	NS	30	16.4 (10.2-25.4)	NS	7	9.9 (4.0-22.6)	NS
Never appropriate for government to take guns	106	13.7 (10.4-17.9)	46	11.3 (7.5-16.6)	.02^c^	45	22.9 (15.2-33.0)	.02^d^	3	9.3 (1.8-36.1)	NS
Worried about retaliation	88	11.3 (8.4-15.0)	60	11.6 (8.2-16.3)	NS	17	9.4 (4.5-18.6)	NS	7	17.0 (6.3-38.5)	NS
Other reason	101	10.7 (7.8-14.4)	59	8.5 (5.8-12.4)	NS	30	18.2 (9.9-31.1)	NS	5	8.7 (2.9-23.3)	NS

Abbreviation: NS, not significant.

^a^ Percentages of respondents who did not endorse each reason or refused to respond are not shown.

^b^ Respondents were asked, “You mentioned that you were not at all willing to ask a judge for a GVRO in 1 or more situations. Please choose the reasons why. Select all that apply.”

^c^ Significant difference from firearm owners.

^d^ Significant difference from nonowners.

Firearm owners were significantly less likely than nonowners (33.9% vs 47.8%; *P* = .04) to endorse not knowing enough about GVROs and significantly more likely than nonowners (27.7% vs 14.5%; *P* = .006) to endorse worry about due process rights, to distrust that the system will be fair (34.0% vs 20.1%; *P* = .02), and the notion that it is never appropriate for the government to take a person’s guns (22.9% vs 11.3%; *P* = .02) as reasons for their unwillingness to use a GVRO (Table 5).

When all respondents, regardless of willingness to ask a judge for a GVRO in specific risk scenarios, were asked whether they would prefer to have the police ask a judge for a GVRO for them, 35.4% (95% CI, 32.8%-38.1%) said yes, 38.6% (95% CI, 35.9%-41.4%) said they did not know, and 24.7% (95% CI, 22.4%-27.1%) said no (eTable 4 in the Supplement). Firearm owners (33.5%) were significantly more likely than both nonowners who live with owners (21.2%; *P* = .02) and nonowners (23.3%; *P* = .004) to report that no, they did not prefer to have the police ask a judge for a GVRO for them and significantly less likely than nonowners to report that they did not know (30.3% vs 39.6%; *P* = .01).

## Discussion

Extreme risk protection orders temporarily suspend access to firearms and ammunition by individuals whom a judge has deemed to be at substantial risk of harming themselves or others. This California-based study documents widespread public support—more than 70% across various risk scenarios—for the use of GVROs (California’s term for ERPOs) at least some of the time, both in general and when the at-risk person is a hypothetical family member. Firearm owners, even those without preexisting awareness of GVROs (results not shown), reported the highest levels of at least some support for the appropriateness of GVROs, in general, in 4 of 5 risk scenarios, while non–gun-owning respondents who live with owners reported the highest levels of at least some willingness to ask a judge for a GVRO for a family member, across all 5 risk scenarios.

Although uptake of ERPOs, especially by non–law-enforcement petitioners, has been slow in California and across the country, these findings suggest that resistance from individuals most likely to be affected by the law (eg, firearm owners, those with social proximity to owners, and individuals who know someone at risk of harm) does not account for such lags. These findings are consistent with national studies^9^ indicating that many firearm violence prevention policies have broad public support, and that general consensus exists between firearm owners and nonowners; although notably, greater support among gun owners vs nonowners in California is unique relative to prior national estimates. These findings also align with expert guidance and past research^16^ that found higher levels of public support, especially among owners, when firearm violence prevention efforts, including firearm safety conversations between health care professionals and patients, are implemented in the context of risk reduction.

Extreme risk protection order laws exemplify a targeted, risk-based approach to preventing harm before it occurs, leveraging the knowledge of those who are often the first to recognize that someone they care about is in crisis or behaving dangerously, and providing a tool for proactive intervention. Studies document that ERPOs have been used in efforts to prevent mass harm,^21^ and that they may be particularly effective in preventing suicide^22^ because access to a firearm during what is often an impulsive act of self-harm^23^ can mean the difference between death and survival. These findings suggest that most adults in California are receptive to this characterization of firearm-related harm as preventable rather than inevitable, and when provided with a mechanism to intervene before violence occurs, a large share of the population—including gun owners and those who live with owners—not only think it is at least sometimes appropriate but are somewhat or very willing to take action personally on behalf of someone who is at increased risk.

Nonetheless, GVRO awareness levels are low. Approximately two-thirds of respondents had never heard of a GVRO or a red flag law, and pluralities of respondents within the subsets of those who did not know whether a GVRO was appropriate (19%) and who were not at all willing to ask a judge for a GVRO (30%) in 1 or more risk scenarios said they needed more information about GVROs. This lack of awareness represents a substantial barrier to GVRO implementation at the population level and presents a clear opportunity for improvement. We recommend further research to assess what additional information would amplify public understanding and use of GVROs and how such messaging can be disseminated in ways that are relevant, accurate, and effective.^24,25^

These findings provide several insights for addressing hesitancy to use GVROs. First, among the subset of those who were not at all willing to ask a judge for a GVRO in 1 or more scenarios of increased risk, the perception that these are personal or family matters was cited by more than 1 of 4 respondents. Similar to the shifts in the public’s understanding of intimate partner violence, it will be important to normalize the process of intervening to reduce an at-risk individual’s access to guns as an issue affecting society as a whole (including mass harm and domestic terrorism), rather than only as a private problem. Second, among firearm owners in particular, it will be essential to address concerns about due process and the notion that it is never appropriate for the government to regulate a person’s access to guns. While the latter may require more sustained investments in shifting cultural paradigms about private firearm ownership, the former may be more readily addressed through targeted messaging efforts to emphasize that ERPO-type laws have built in due process protections, including a timely hearing before a judge at which petitioners must meet a standard of proof and counter evidence can be presented.^24^

Finally, it will also be necessary to address distrust of the mainstream criminal legal system, which has systematically oppressed and disenfranchised Asian, Black, and Latinx communities through historical and present policies and practices. More than 20% of respondents who were not at all willing to use a GVRO in 1 or more risk scenarios cited distrust in the fairness of the system and 16% said they did not want to involve the court. A separate analysis that is not yet published will examine whether responses fell along any specific or expected racial-ethnic lines. As recently argued,^26^ it will be essential to include assessment of social inequities associated with ERPO implementation and future evaluation—including possible consequences of unequal enforcement and benefits of reduced harm—so that the use of these tools does not perpetuate the uneven burden of structurally rooted trauma and injustice within marginalized communities.

### Limitations

This study has several limitations. First, we relied on self-reported data, which is subject to nonresponse and social desirability biases; however, nonresponse rates were low in the study sample, and evidence suggests that completion rates are higher and social desirability bias is lower in online panel surveys than in random-digit-dial telephone surveys.^27^ Second, although we think these findings can inform understandings of EPRO implementation in other states, California is unique. Geographic variations in firearm ownership and the cultural acceptability of firearms, firearm violence, and firearm regulations should be considered when attempting to generalize these results. Third, primary analyses that collapse response categories (eg, somewhat, usually, and always appropriate; somewhat and very willing) may mask more nuanced differences in the extent of support for GVRO appropriateness and willingness overall and by firearm ownership status. However, given the polarizing nature of gun policy discourse, we posit that any indication of support for the appropriateness of or willingness to use an EPRO is not only noteworthy, but may also be valuable for changing behavior via subjective norms—ie, beliefs that an important person or group of people will approve and support a particular behavior. Finally, although we measured the public’s perceived appropriateness of GVROs in general and their personal willingness to use these tools, the extent to which such support translates into real-world action is unclear.

## Conclusions

This cross-sectional study found that ERPOs are promising tools for reducing firearm-related harm. Although we found widespread public support (including among firearm owners and nonowners who live with owners) for using ERPOs at least some of the time across a range of risk scenarios, public awareness levels are low. Attention to concerns regarding unjust implementation of ERPOs is warranted. This study identifies concrete areas of focus for efforts to address ERPO uptake and implementation challenges in California and other states with similar laws in effect or under consideration.

## Notes

Supplement. eMethods. Detailed question wording and response options, 2020 California Safety and Wellbeing Survey eTable 1A. Perceived appropriateness of a judge issuing a GVRO, in general, by risk scenario and firearm ownership status, 2020 California Safety and Wellbeing Survey eTable 1B. Willingness to ask a judge for a GVRO for a family member, by risk scenario and firearm ownership status, 2020 California Safety and Wellbeing Survey eTable 2. Need more information about GVROs, respondents who did not know if a GVRO is appropriate in ≥1 risk scenarios (n = 449) eTable 3A. Willingness to ask a judge for a GVRO for a family member who has threatened to physically harm someone else, by number of known others who have been shot by someone else on purpose eTable 3B. Willingness to ask a judge for a GVRO for a family member who has threatened to physically harm a group of people, by number of known others who have been shot by someone else on purpose eTable 3C. Willingness to ask a judge for a GVRO for a family member who has threatened to physically harm themselves, by number of known others who have shot themselves on purpose eTable 3D. Willingness to ask a judge for a GVRO for a family member who has threatened to physically harm someone else, by concern a known other might physically hurt another person on purpose eTable 3E. Willingness to ask a judge for a GVRO for a family member who has threatened to physically harm a group of people, by concern a known other might physically hurt another person on purpose eTable 3F. Willingness to ask a judge for a GVRO for a family member who has threatened to physically harm themselves, by concern a known other might physically hurt themselves on purpose eTable 4. Prefer to have the police ask a judge for a GVRO for you, by firearm ownership status (n = 2870)

## References

[1] US Centers for Disease Control and Prevention. Fatal injury reports. Web-based Injury Statistics Query and Analysis System (WISQARS). Accessed August 31, 2020. https://www.cdc.gov/injury/wisqars/index.html

[2] Rivara FP, Vars FE, Rowhani-Rahbar A. Three interventions to address the other pandemic–firearm injury and death. JAMA. 2021;325(4):343-344. doi:10.1001/jama.2020.24206 33416855

[3] Laqueur HS, Wintemute GJ. Identifying high-risk firearm owners to prevent mass violence. Criminol Public Policy. 2020;19(1):109-127. doi:10.1111/1745-9133.12477

[4] Pane LM. Parkland attack fueled big shift in America’s gun politics. Associated Press. Accessed February 7, 2019. https://apnews.com/article/7d124912b2ff43ce8952009e4c326aea

[5] Giffords Law Center to Prevent Gun Violence. Who can have a gun: extreme risk protection orders. Accessed February 1, 2021. https://giffords.org/lawcenter/gun-laws/policy-areas/who-can-have-a-gun/extreme-risk-protection-orders/

[6] Bloomberg American Health Initiative. Extreme risk protection order: a tool to save lives. Accessed February 1, 2021. https://americanhealth.jhu.edu/implementERPO

[7] Pallin R, Schleimer JP, Pear VA, Wintemute GJ. Assessment of extreme risk protection order use in California from 2016 to 2019. JAMA Netw Open. 2020;3(6):e207735-e207735. doi:10.1001/jamanetworkopen.2020.7735 32556258 PMC7303810

[8] Rowhani-Rahbar A, Bellenger MA, Gibb L, . Extreme risk protection orders in Washington: a statewide descriptive study. Ann Intern Med. 2020;173(5):342-349. doi:10.7326/M20-0594 32598226

[9] Barry CL, Webster DW, Stone E, Crifasi CK, Vernick JS, McGinty EE. Public support for gun violence prevention policies among gun owners and non-gun owners in 2017. Am J Public Health. 2018;108(7):878-881. doi:10.2105/AJPH.2018.304432 29771617 PMC5993389

[10] Martin DD, Wyatt KL, Shanahan SB. Practitioners’ perspective on extreme risk protection orders. JAMA Netw Open. 2020;3(6):e208021-e208021. doi:10.1001/jamanetworkopen.2020.8021 32556254

[11] Kravitz-Wirtz N, Aubel A, Schleimer J, Pallin R, Wintemute G. Public concern about violence, firearms, and the COVID-19 pandemic in California. JAMA Netw Open. 2021;4(1):e2033484-e2033484. doi:10.1001/jamanetworkopen.2020.33484 33394004 PMC7783542

[12] Anestis MD, Bond AE, Daruwala SE, Bandel SL, Bryan CJ. Suicidal ideation among individuals who have purchased firearms during COVID-19. Am J Prev Med. 2021;60(3):311-317. doi:10.1016/j.amepre.2020.10.013 33358551

[13] Lyons VH, Haviland MJ, Azrael D, . Firearm purchasing and storage during the COVID-19 pandemic. Inj Prev. 2021;27(1):87-92. doi:10.1136/injuryprev-2020-043872 32943492

[14] Galea S, Abdalla SM. COVID-19 pandemic, unemployment, and civil unrest: underlying deep racial and socioeconomic divides. JAMA. 2020;324(3):227-228. doi:10.1001/jama.2020.11132 32530457

[15] Pollack CE, Leifheit KM, Linton SL. When storms collide: evictions, COVID-19, and health equity. Health Affairs Blog. Accessed April 26, 2021. https://www.healthaffairs.org/do/10.1377/hblog20200730.190964/full/

[16] Pallin R, Charbonneau A, Wintemute GJ, Kravitz-Wirtz N. California public opinion on health professionals talking with patients about firearms. Health Aff (Millwood). 2019;38(10):1744-1751. doi:10.1377/hlthaff.2019.00602 31589535

[17] Betz ME, Azrael D, Johnson RL, . Views on firearm safety among caregivers of people with Alzheimer disease and related dementias. JAMA Netw Open. 2020;3(7):e207756-e207756. doi:10.1001/jamanetworkopen.2020.7756 32667652 PMC7364369

[18] Betz ME, Azrael D, Barber C, Miller M. Public opinion regarding whether speaking with patients about firearms is appropriate: results of a national survey. Ann Intern Med. 2016;165(8):543-550. doi:10.7326/M16-0739 27455516

[19] Pham-Kanter G, Mello MM, Lehmann LS, Campbell EG, Carpenter D. Public awareness of and contact with physicians who receive industry payments: a national survey. J Gen Intern Med. 2017;32(7):767-774. doi:10.1007/s11606-017-4012-3 28265803 PMC5481229

[20] US Census Bureau. American Community Survey 2108. Accessed April 30, 2021. https://www.census.gov/programs-surveys/acs

[21] Wintemute GJ, Pear VA, Schleimer JP, . Extreme risk protection orders intended to prevent mass shootings: a case series. Ann Intern Med. 2019;171(9):655-658. doi:10.7326/M19-2162 31426088

[22] Swanson J, Norko M, Lin H-J, . Implementation and effectiveness of Connecticut’s risk-based gun removal law: does it prevent suicides? Law Contemp Probl. 2017;80:179-208. https://scholarship.law.duke.edu/lcp/vol80/iss2/8

[23] Owens D, Horrocks J, House A. Fatal and non-fatal repetition of self-harm: systematic review. Br J Psychiatry. 2002;181(3):193-199. doi:10.1192/bjp.181.3.193 12204922

[24] Giffords, Educational Fund to Stop Gun Violence, Alliance for Gun Responsibility. Extreme risk laws: a toolkit for developing life-saving policy in your state. Accessed April 26, 2021. https://giffords.org/wp-content/uploads/2018/08/Extreme-Risk-Laws-Toolkit.pdf

[25] Consortium for Risk-Based Firearm Policy. Extreme risk protection orders: new recommendations for policy and implementation. Accessed April 26, 2021. https://efsgv.org/wp-content/uploads/EFSGV-ConsortiumReport2020-ERPOs.pdf

[26] Swanson JW. The color of risk protection orders: gun violence, gun laws, and racial justice. Inj Epidemiol. 2020;7(1):46. doi:10.1186/s40621-020-00272-z 32772923 PMC7416407

[27] Kreuter F, Presser S, Tourangeau R. Social desirability bias in CATI, IVR, and web surveys: the effects of mode and question sensitivity. Public Opinion Quarterly. 2008;72(5):847-865. doi:10.1093/poq/nfn063

