# Supplementary material for: Public Awareness of and Personal Willingness to Use California's Extreme Risk Protection Order Law to Prevent Firearm-Related Harm
Source: JAMA Health Forum. 2021 Jun 4;2(6):e210975. doi: 10.1001/jamahealthforum.2021.0975 (PMC8796972; doi:10.1001/jamahealthforum.2021.0975)
Supplement: Supplement. — eMethods. Detailed question wording and response options, 2020 California Safety and Wellbeing Survey eTable 1A. Perceived appropriateness of a judge issuing a GVRO, in general, by risk scenario and firearm ownership status, 2020 California Safety and Wellbeing Survey eTable 1B. Willingness to ask a judge for a GVRO for a family member, by risk scenario and firearm ownership status, 2020 California Safety and Wellbeing Survey eTable 2. Need more information about GVROs, respondents who did not know if a GVRO is appropriate in ≥1 risk scenarios (n = 449) eTable 3A. Willingness to ask a judge for a GVRO for a family member who has threatened to physically harm someone else, by number of known others who have been shot by someone else on purpose eTable 3B. Willingness to ask a judge for a GVRO for a family member who has threatened to physically harm a group of people, by number of known others who have been shot by someone else on purpose eTable 3C. Willingness to ask a judge for a GVRO for a family member who has threatened to physically harm themselves, by number of known others who have shot themselves on purpose eTable 3D. Willingness to ask a judge for a GVRO for a family member who has threatened to physically harm someone else, by concern a known other might physically hurt another person on purpose eTable 3E. Willingness to ask a judge for a GVRO for a family member who has threatened to physically harm a group of people, by concern a known other might physically hurt another person on purpose eTable 3F. Willingness to ask a judge for a GVRO for a family member who has threatened to physically harm themselves, by concern a known other might physically hurt themselves on purpose eTable 4. Prefer to have the police ask a judge for a GVRO for you, by firearm ownership status (n = 2870) [file jamahealthforum-e210975-s001.pdf]

# Supplemental Online Content

Kravitz-Wirtz N, Aubel AJ, Pallin R, Wintemute GJ. Public awareness of and personal willingness to use California's extreme risk protection order law to prevent firearm-related harm. *JAMA Health Forum*. 2021;2(6):e210975. doi:10.1001/jamahealthforum.2021.0975

**eMethods.** Detailed question wording and response options, 2020 California Safety and Wellbeing Survey
**eTable 1A.** Perceived appropriateness of a judge issuing a GVRO, in general, by risk scenario and firearm ownership status, 2020 California Safety and Wellbeing Survey
**eTable 1B.** Willingness to ask a judge for a GVRO for a family member, by risk scenario and firearm ownership status, 2020 California Safety and Wellbeing Survey
**eTable 2.** Need more information about GVROs, respondents who did not know if a GVRO is appropriate in ≥1 risk scenarios (n = 449)
**eTable 3A.** Willingness to ask a judge for a GVRO for a family member who has threatened to physically harm someone else, by number of known others who have been shot by someone else on purpose
**eTable 3B.** Willingness to ask a judge for a GVRO for a family member who has threatened to physically harm a group of people, by number of known others who have been shot by someone else on purpose
**eTable 3C**. Willingness to ask a judge for a GVRO for a family member who has threatened to physically harm themselves, by number of known others who have shot themselves on purpose
**eTable 3D.** Willingness to ask a judge for a GVRO for a family member who has threatened to physically harm someone else, by concern a known other might physically hurt another person on purpose
**eTable 3E.** Willingness to ask a judge for a GVRO for a family member who has threatened to physically harm a group of people, by concern a known other might physically hurt another person on purpose
**eTable 3F.** Willingness to ask a judge for a GVRO for a family member who has threatened to physically harm themselves, by concern a known other might physically hurt themselves on purpose
**eTable 4.** Prefer to have the police ask a judge for a GVRO for you, by firearm ownership status (n = 2870)

This supplemental material has been provided by the authors to give readers additional information about their work.

© 2021 Kravitz-Wirtz et al. *JAMA Health Forum.*

eMethods. Detailed question wording and response options, 2020 California Safety and Wellbeing Survey

Have you ever heard of something called a gun violence restraining order?
1. Yes
2. No

Have you ever heard of something called a "red flag" law?
1. Yes
2. No

IntroG_1.
California has something called a gun violence restraining order or GVRO. These are also sometimes called "red flag" laws. When someone is threatening to hurt themselves or someone else, and they have or could get a gun, a GVRO can be used to temporarily prevent that person from having or buying guns.

The person's immediate family, household members, or the police can ask a judge to give this order. In an emergency, the judge can issue an order immediately that lasts for up to 3 weeks. After a court hearing the judge can issue an order that lasts for up to 1 year.

GVROs are only available if other options to protect against harm have failed or are not appropriate.

[RANDOMIZE AND DISPLAY 1 OF THE 4 FOLLOWING: ]
A. [Scripter: No statement shown for this random group]
B. Research suggests that GVROs prevent violence.
C. Research suggests that GVROs prevent violence. There have been more than 20 cases in California where GVROs were used in an effort to prevent mass shootings. Those mass shootings did not occur.
D. Research suggests that GVROs prevent violence. In other states, studies have found that 1 life is saved for every 10 to 20 GVROs used to prevent suicide.

In general, do you think it would be appropriate for a judge to issue a GVRO in the following scenarios? Assume the person has or could get a gun and other options have failed or are not appropriate.

Statements per row:
1. The person is experiencing an emotional crisis
2. The person has severe dementia or something like it
3. The person has threatened to physically hurt themselves
4. The person has threatened to physically hurt you or someone else
5. The person has threatened to physically hurt a group of people

Answers in columns:
1. Never appropriate
2. Sometimes appropriate
3. Usually appropriate
4. Always appropriate
5. Don't know

You mentioned that you didn't know. Is this because you need more information about GVROs?
1. Yes
2. No

Would <u>you personally</u> be willing to ask a judge for a GVRO if a member of your family was in one of the following scenarios? Assume your family member has or could get a gun and other options have failed or are not appropriate.

© 2021 Kravitz-Wirtz et al. *JAMA Health Forum.*

Statements per row:
1. They were experiencing an emotional crisis
2. They had severe dementia or something like it
3. They had threatened to physically hurt themselves
4. They had threatened to physically hurt you or someone else
5. They had threatened to physically hurt a group of people

Answers in columns:
1. Not at all willing
2. Somewhat willing
3. Very willing

Would you prefer to have the police ask a judge for a GVRO for you?
1. Yes
2. No
3. Don't know

You mentioned that you were not at all willing to ask a judge for a GVRO in one or more situations. Please choose the reasons why. Select all that apply.
1. I don't know enough about GVROs
2. I'm worried about retaliation
3. I'm worried about due-process rights
4. I don't want to involve the court
5. I don't trust the system to be fair
6. These are personal or family matters
7. It is never appropriate for the government to take a person's guns
8. Other, please specify:[Text box]

Not counting yourself, how many people that you personally know have ever been shot by someone else? Do not count people in the armed forces who were shot in the line of duty.
[Dropdown 0-25]

Was this an accident or on purpose?
1. An accident
2. On purpose
3. Don't know

You said you personally know [insert number] people who have been shot by someone else. How many of these people were shot by accident?
[Dropdown 0-25]

How many of these people were shot on purpose?
[Dropdown 0-25]

Not counting yourself, how many people that you personally know have ever shot themselves?

Was this an accident or on purpose?
1. An accident
2. On purpose
3. Don't know

You said you personally know [insert number] people who have shot themselves. How many of these people shot themselves by accident?

© 2021 Kravitz-Wirtz et al. *JAMA Health Forum.*

[Dropdown 0-25]

How many of these people shot themselves on purpose?
[Dropdown 0-25]

Are you concerned that anyone you know might physically hurt another person on purpose? Consider only people you know personally, not people you've only heard about from others or seen in the media.
1. Yes
2. No

Are you concerned that anyone you know might physically hurt themselves on purpose? Consider only people you know personally, not people you've only heard about from others or seen in the media.
1. Yes
2. No

© 2021 Kravitz-Wirtz et al. *JAMA Health Forum.*

eTable 1A. Perceived Appropriateness of a Judge Issuing a GVRO, In General, by Risk Scenario and Firearm Ownership Status, 2020 California Safety and Wellbeing Survey

| | | | | | | | Emotional crisis | | | | | | | | |
|---|---|---|---|---|---|---|---|---|---|---|---|---|---|---|---|
| | Never | | | Sometimes | | | Usually | | | Always | | | Don't know | | |
| | n | % | 95% CI | n | % | 95% CI | n | % | 95% CI | n | % | 95% CI | n | % | 95% CI |
| Non-owners | 182 | 11.3 | 9.3-13.7 | 732 | 31.8 | 28.8-34.9 | 396 | 19.3 | 16.8-22.0 | 418 | 21.2 | 18.6-24.0 | 246 | 15.5 | 13.1-18.2 |
| Firearm owners | 76 | 15.0 | 10.9-20.3 | 251 | 42.0 | 35.6-48.7 | 89 | 19.9 | 14.7-26.5 | 70 | 13.3 | 9.4-18.4 | 42 | 9.7 | 6.1-15.0 |
| Non-owners who live with owners | 15 | 6.7 | 2.9-14.7 | 96 | 35.0 | 26.7-44.3 | 38 | 25.3 | 16.9-35.9 | 44 | 21.9 | 15.0-30.9 | 26 | 11.1 | 6.7-17.8 |
| TOTAL | 296 | 11.5 | 9.9-13.5 | 1123 | 33.6 | 31.0-36.2 | 538 | 19.9 | 17.7-22.2 | 542 | 19.5 | 17.4-21.8 | 346 | 14.4 | 12.5-16.6 |
| | | | | | | | Severe dementia or something like it | | | | | | | | |
| | Never | | | Sometimes | | | Usually | | | Always | | | Don't know | | |
| | n | % | 95% CI | n | % | 95% CI | n | % | 95% CI | n | % | 95% CI | n | % | 95% CI |
| Non-owners | 198 | 12.3 | 10.2-14.8 | 382 | 16.5 | 14.3-19.0 | 369 | 18.1 | 15.7-20.8 | 808 | 37.1 | 34.0-40.4 | 201 | 13.8 | 11.4-16.5 |
| Firearm owners | 50 | 10.7 | 7.2-15.5 | 127 | 21.2 | 16.4-26.8 | 127 | 23.1 | 17.8-29.4 | 186 | 35.7 | 29.5-42.4 | 36 | 9.1 | 5.5-14.9 |
| Non-owners who live with owners | 19 | 13.4 | 7.1-23.6 | 46 | 19.4 | 13.3-27.6 | 44 | 16.3 | 10.5-24.5 | 90 | 40.5 | 31.3-50.4 | 20 | 10.4 | 6.0-17.3 |
| TOTAL | 286 | 12.2 | 10.4-14.2 | 578 | 17.4 | 15.5-19.5 | 561 | 19.0 | 16.9-21.3 | 1114 | 36.5 | 33.9-39.2 | 288 | 13.0 | 11.1-15.1 |
| | | | | | | | Threatened to physically harm themselves | | | | | | | | |
| | Never | | | Sometimes | | | Usually | | | Always | | | Don't know | | |
| | n | % | 95% CI | n | % | 95% CI | n | % | 95% CI | n | % | 95% CI | n | % | 95% CI |
| Non-owners | 162 | 11.2 | 9.2-13.7 | 226 | 10.1 | 8.4-12.2 | 414 | 20.5 | 17.9-23.4 | 1021 | 46.3 | 43.0-49.6 | 138 | 10.2 | 8.1-12.7 |
| Firearm owners | 40 | 11.8 | 7.8-17.4 | 84 | 12.9 | 9.2-17.8 | 142 | 24.7 | 19.4-30.9 | 240 | 46.5 | 39.8-53.3 | 22 | 4.0 | 2.3-6.8 |
| Non-owners who live with owners | 16 | 9.4 | 4.5-18.8 | 16 | 4.5 | 2.4-8.3 | 54 | 25.1 | 17.4-34.7 | 117 | 51.1 | 41.4-60.7 | 15 | 9.6 | 5.2-17.0 |
| TOTAL | 235 | 11.3 | 9.5-13.3 | 343 | 10.1 | 8.6-11.8 | 640 | 22.1 | 19.8-24.6 | 1413 | 45.7 | 42.9-48.4 | 200 | 9.3 | 7.7-11.2 |
| | | | | | | | Threatened to physically harm someone else | | | | | | | | |
| | Never | | | Sometimes | | | Usually | | | Always | | | Don't know | | |
| | n | % | 95% CI | n | % | 95% CI | n | % | 95% CI | n | % | 95% CI | n | % | 95% CI |
| Non-owners | 169 | 11.9 | 9.8-14.4 | 156 | 7.2 | 5.8-9.0 | 304 | 15.6 | 13.3-18.2 | 1202 | 53.8 | 50.4-57.1 | 127 | 9.2 | 7.2-11.6 |
| Firearm owners | 36 | 9.5 | 6.2-14.3 | 55 | 9.4 | 6.3-13.8 | 117 | 19.1 | 14.5-24.8 | 302 | 57.0 | 50.3-63.5 | 16 | 3.4 | 1.8-6.2 |
| Non-owners who live with owners | 15 | 8.9 | 4.1-18.4 | 9 | 4.0 | 1.7-8.9 | 38 | 17.2 | 10.7-26.5 | 143 | 62.2 | 52.1-71.3 | 14 | 7.7 | 4.0-14.3 |
| TOTAL | 235 | 11.3 | 9.5-13.3 | 236 | 7.4 | 6.1-8.8 | 484 | 16.6 | 14.6-18.8 | 1693 | 54.2 | 51.4-57.0 | 177 | 8.2 | 6.7-10.1 |
| | | | | | | | Threatened to physically harm a group of people | | | | | | | | |
| | Never | | | Sometimes | | | Usually | | | Always | | | Don't know | | |
| | n | % | 95% CI | n | % | 95% CI | n | % | 95% CI | n | % | 95% CI | n | % | 95% CI |
| Non-owners | 172 | 12.4 | 10.2-15.0 | 113 | 5.2 | 3.9-6.8 | 262 | 14.0 | 11.8-16.5 | 1294 | 58.4 | 55.0-61.7 | 122 | 8.4 | 6.5-10.7 |
| Firearm owners | 36 | 9.8 | 6.2-15.2 | 40 | 6.3 | 4.0-9.8 | 97 | 16.1 | 11.7-21.7 | 334 | 62.9 | 56.2-69.1 | 19 | 4.4 | 2.4-7.8 |
| Non-owners who live with owners | 15 | 9.3 | 4.4-18.7 | 8 | 2.6 | 1.2-6.0 | 28 | 14.2 | 8.2-23.6 | 154 | 64.7 | 54.4-73.7 | 14 | 9.2 | 4.9-16.5 |
| TOTAL | 238 | 11.7 | 9.9-13.8 | 173 | 5.2 | 4.2-6.5 | 410 | 14.6 | 12.6-16.7 | 1832 | 58.7 | 55.9-61.4 | 178 | 8.1 | 6.6-9.9 |

Respondents were asked, "In general, do you think it would be appropriate for a judge to issue a GVRO in the following scenarios?"

Percentages may not sum to 100% because refusals are not shown.

N

© 2021 Kravitz-Wirtz et al. *JAMA Health Forum.*

eTable 1B. Willingness to Ask a Judge for a GVRO for a Family Member, by Risk Scenario and Firearm Ownership Status, 2020 California Safety and Wellbeing Survey

| | Emotional crisis | | | | | | | | |
|---|---|---|---|---|---|---|---|---|---|
| | Not at all | | | Somewhat | | | Very | | |
| | n | % | 95% CI | n | % | 95% CI | n | % | 95% CI |
| Non-owners | 406 | 24.2 | 21.3-27.3 | 917 | 42.2 | 38.9-45.4 | 631 | 30.9 | 28.0-34.1 |
| Firearm owners | 161 | 29.8 | 23.9-36.4 | 243 | 47.8 | 41.1-54.6 | 122 | 22 | 17.5-27.3 |
| Non-owners who live with owners | 38 | 16.3 | 10.0-25.3 | 111 | 50.8 | 41.1-60.5 | 70 | 32.9 | 24.6-42.5 |
| TOTAL | 651 | 24.4 | 22.0-27.0 | 1325 | 44.1 | 41.4-46.9 | 843 | 29 | 26.6-31.6 |
| | Severe dementia or something like it | | | | | | | | |
| | Not at all | | | Somewhat | | | Very | | |
| | n | % | 95% CI | n | % | 95% CI | n | % | 95% CI |
| Non-owners | 348 | 20.9 | 18.2-23.9 | 632 | 30.7 | 27.7-33.8 | 965 | 45.4 | 42.1-48.7 |
| Firearm owners | 103 | 21.5 | 16.3-27.8 | 187 | 35 | 28.7-41.9 | 234 | 43 | 36.5-49.7 |
| Non-owners who live with owners | 25 | 15.3 | 8.9-25.0 | 71 | 33.3 | 24.6-43.3 | 122 | 51.2 | 41.5-60.9 |
| TOTAL | 515 | 20.6 | 18.4-23.1 | 929 | 31.4 | 28.9-34.0 | 1361 | 45.1 | 42.3-47.8 |
| | Threatened to physically harm themselves | | | | | | | | |
| | Not at all | | | Somewhat | | | Very | | |
| | n | % | 95% CI | n | % | 95% CI | n | % | 95% CI |
| Non-owners | 207 | 14.3 | 11.9-17.0 | 467 | 22.5 | 19.8-25.3 | 1273 | 59.9 | 56.6-63.2 |
| Firearm owners | 68 | 16.6 | 11.9-22.6 | 137 | 23 | 17.7-29.4 | 321 | 60 | 53.1-66.5 |
| Non-owners who live with owners | 9 | 5.4 | 1.9-14.5 | 56 | 30 | 21.6-40.0 | 154 | 64.6 | 54.3-73.7 |
| TOTAL | 318 | 14.2 | 12.3-16.5 | 707 | 23.9 | 21.6-26.4 | 1786 | 58.9 | 56.1-61.6 |
| | Threatened to physically harm someone else | | | | | | | | |
| | Not at all | | | Somewhat | | | Very | | |
| | n | % | 95% CI | n | % | 95% CI | n | % | 95% CI |
| Non-owners | 188 | 14.1 | 11.7-16.9 | 363 | 18.3 | 15.9-21.0 | 1395 | 64.3 | 61.0-67.5 |
| Firearm owners | 57 | 14.2 | 9.8-20.1 | 110 | 15.3 | 11.3-20.5 | 360 | 70.2 | 63.7-76.0 |
| Non-owners who live with owners | 9 | 5.1 | 1.8-13.8 | 42 | 22.0 | 14.9-31.2 | 167 | 72.8 | 62.9-80.8 |
| TOTAL | 280 | 13.4 | 11.4-15.6 | 560 | 19.2 | 17.1-21.5 | 1969 | 64.5 | 61.8-67.2 |
| | Threatened to physically harm a group of people | | | | | | | | |
| | Not at all | | | Somewhat | | | Very | | |
| | n | % | 95% CI | n | % | 95% CI | n | % | 95% CI |
| Non-owners | 192 | 14.4 | 12.0-17.2 | 316 | 15.8 | 13.5-18.3 | 1445 | 66.8 | 63.5-69.9 |
| Firearm owners | 55 | 14 | 9.6-20.0 | 98 | 17.4 | 12.5-23.6 | 372 | 68 | 61.0-74.2 |
| Non-owners who live with owners | 11 | 7 | 2.9-15.8 | 37 | 18 | 11.7-26.8 | 171 | 75 | 65.2-82.8 |
| TOTAL | 284 | 13.7 | 11.7-15.9 | 488 | 17 | 15.0-19.2 | 2045 | 66.6 | 63.8-69.3 |

Respondents were asked, "Would you personally be willing to ask a judge for a GVRO if a member of your family was in one of the following scenarios?"

Percentages may not sum to 100% because refusals are not shown.

© 2021 Kravitz-Wirtz et al. *JAMA Health Forum.*

eTable 2. Need More Information about GVROs, Respondents Who Did Not Know if a GVRO is Appropriate in ≥1 Risk Scenarios (n=449)

| | n | % | 95 % CI |
|---|---|---|---|
| Yes | 196 | 48.5 | 41.8-55.3 |
| No | 247 | 49.2 | 42.4-55.9 |

Respondents were asked, "You mentioned that you sometimes didn’t know. Is this because you need more information about GVROs?" Percentages may not sum to 100% because refusals are not shown.

© 2021 Kravitz-Wirtz et al. *JAMA Health Forum.*

eTable 3A. Willingness to Ask a Judge for a GVRO for a Family Member who has Threatened to Physically Harm Someone Else, by Number of Known Others who have been Shot by Someone Else on Purpose

| | Not at all | | | Somewhat/Very | | | Total |
|---|---|---|---|---|---|---|---|
| | n | % | 95% CI | n | % | 95% CI | n |
| 0 | 188 | 12.4 | 10.2-15.0 | 1927 | 85.7 | 82.9-88.0 | 2146 |
| 1+ | 63 | 14.0 | 10.0-19.3 | 504 | 85.6 | 80.4-89.7 | 572 |
| Total | 280 | 13.4 | 11.4-15.6 | 2529 | 83.8 | 81.4-85.9 | 2870 |

eTable 3B. Willingness to Ask a Judge for a GVRO for a Family Member who has Threatened to Physically Harm a Group of People, by Number of Known Others who have been Shot by Someone Else on Purpose

| | Not at all | | | Somewhat/Very | | | Total |
|---|---|---|---|---|---|---|---|
| | n | % | 95% CI | n | % | 95% CI | n |
| 0 | 194 | 12.7 | 10.5-15.3 | 1926 | 85.4 | 82.7-87.8 | 2146 |
| 1+ | 61 | 14.5 | 10.4-19.9 | 510 | 85.4 | 80.0-89.5 | 572 |
| Total | 284 | 13.7 | 11.7-15.9 | 2533 | 83.6 | 81.2-85.8 | 2870 |

Respondents were asked, "Not counting yourself, how many people that you personally know have ever been shot by someone else? Do not count people in the armed forces who were shot in the line of duty" and "How many of these people were shot on purpose?"

eTable 3C. Willingness to Ask a Judge for a GVRO for a Family Member who has Threatened to Physically Harm Themselves, by Number of Known Others who have Shot Themselves on Purpose

| | Not at all | | | Somewhat/Very | | | Total | |
|---|---|---|---|---|---|---|---|---|
| | n | % | 95% CI | n | % | 95% CI | n | |
| 0 | 259 | 14.8 | 12.5-17.4 | 1925 | 83.0 | 80.3-85.4 | 2219 | |
| 1+ | 40 | 9.2 | 6.1-13.6 | 506 | 90.6 | 86.2-93.8 | 547 | ** |
| Total | 318 | 14.2 | 12.3-16.5 | 2493 | 82.8 | 80.4-85.0 | 2870 | |

Respondents were asked, "Not counting yourself, how many people that you personally know have ever shot themselves?" and "How many of these people shot themselves on purpose?"

*p<0.05 **p<0.01

© 2021 Kravitz-Wirtz et al. *JAMA Health Forum.*

eTable 3D. Willingness to Ask a Judge for a GVRO for a Family Member who has Threatened to Physically Harm Someone Else, by Concern a Known Other Might Physically Hurt Another Person on Purpose

| | Not at all | | | Somewhat/Very | | | Total |
|---|---|---|---|---|---|---|---|
| | n | % | 95% CI | n | % | 95% CI | n |
| Yes | 39 | 13.3 | 8.7-19.8 | 289 | 85.4 | 78.9-90.1 | 335 |
| No | 241 | 13.5 | 11.4-15.9 | 2240 | 84.0 | 81.4-86.3 | 2528 |
| Total | 280 | 13.4 | 11.4-15.6 | 2529 | 83.8 | 81.4-85.9 | 2870 |

eTable 3E. Willingness to Ask a Judge for a GVRO for a Family Member who has Threatened to Physically Harm a Group of People, by Concern a Known Other Might Physically Hurt Another Person on Purpose

| | Not at all | | | Somewhat/Very | | | Total |
|---|---|---|---|---|---|---|---|
| | n | % | 95% CI | n | % | 95% CI | n |
| Yes | 42 | 18.2 | 12.6-25.5 | 288 | 80.8 | 73.4-86.5 | 335 |
| No | 242 | 13.1 | 11.1-15.6 | 2245 | 84.5 | 81.9-86.7 | 2528 |
| Total | 284 | 13.7 | 11.7-15.9 | 2533 | 83.6 | 81.2-85.8 | 2870 |

Respondents were asked, "Are you concerned that anyone you know might physically hurt another person on purpose? Consider only people you know personally, not people you've only heard about from others or seen in the media."

eTable 3F. Willingness to Ask a Judge for a GVRO for a Family Member who has Threatened to Physically Harm Themselves, by Concern a Known Other Might Physically Hurt Themselves on Purpose

| | Not at all | | | Somewhat/Very | | | Total | |
|---|---|---|---|---|---|---|---|---|
| | n | % | 95% CI | n | % | 95% CI | n | |
| Yes | 27 | 9.4 | 5.6-15.4 | 363 | 90.0 | 84.1-93.9 | 395 | ** |
| No | 291 | 15.0 | 12.9-17.5 | 2129 | 82.0 | 79.4-84.3 | 2469 | |
| Total | 318 | 14.2 | 12.3-16.5 | 2493 | 82.8 | 80.4-85.0 | 2870 | |

Respondents were asked, "Are you concerned that anyone you know might physically hurt themselves on purpose? Consider only people you know personally, not people you've only heard about from others or seen in the media."

*p<0.05 **p<0.01

© 2021 Kravitz-Wirtz et al. *JAMA Health Forum.*

eTable 4. Prefer to have the Police Ask a Judge for a GVRO for You, by Firearm Ownership Status (n=2870)

| | Yes | | | | No | | | | Don't know | | | |
|---|---|---|---|---|---|---|---|---|---|---|---|---|
| | n | % | 95% CI | *p* | n | % | 95% CI | *p* | n | % | 95% CI | *p* |
| Non-owners | 731 | 36.0 | 32.9-39.3 | - | 488 | 23.2 | 20.5-26.0 | (2)** | 752 | 39.6 | 36.3-42.9 | (2)** |
| Firearm owners | 193 | 35.8 | 29.5-42.5 | - | 169 | 33.5 | 27.4-40.3 | (1)**(3)* | 166 | 30.3 | 24.7-36.7 | (1)** |
| Non-owners who live with owners | 87 | 42.3 | 32.9-52.3 | - | 51 | 21.2 | 14.6-29.7 | (2)* | 81 | 36.5 | 27.7-46.3 | - |
| TOTAL | 1037 | 35.4 | 32.8-38.1 | - | 743 | 24.7 | 22.4-27.1 | - | 1064 | 38.6 | 35.9-41.4 | - |

Respondents were asked, "Would you prefer to have the police ask a judge for a GVRO for you?"

(1) Significant difference from non-owners
(2) Significant difference from firearm owners
(3) Significant difference from non-owners who live with owners
* p<0.05
** p<0.01

© 2021 Kravitz-Wirtz et al. *JAMA Health Forum.*
